# Surgical management of acute compartment syndrome and sequential complications

**DOI:** 10.1186/s12891-019-2476-5

**Published:** 2019-03-04

**Authors:** Weili Du, Xiaohua Hu, Yuming Shen, Xing Teng

**Affiliations:** 1 Department of Burns, Beijing Jishuitan Hospital, 31 Xinjiekou East Rd, Beijing, 100035 China; 2 Department of Traumatic Orthopedics, Beijing Jishuitan Hospital, Beijing, 100035 China

**Keywords:** Lower leg compartment syndrome, Foot and leg deformity, Wound treatment, Complications

## Abstract

**Background:**

Acute compartment syndrome occurs when pressure within a compartment increases and affects the function of the muscle and tissues after an injury. Compartment syndrome is most common in lower leg and may lead to permanent injury to the muscle and nerves if left untreated.

**Methods:**

46 patients with acute compartment syndrome were enrolled, including 8 cases with serious complications, between January 2008 and December 2012. The protocols combining early management and the correction of deformities were adjusted in order to attempt to enable full recovery of all patients.

**Results:**

All patients had necrotic muscles and nerves, damaged vascular, and severe foot deformities. In the early stage, each patient received systemic support and wound debridement to promote wound healing. For patients with serious complications, a number of medical measures, including installation of Ilizarov external frames, arthrodesis, osteotomy fusion, arthroplasty, or tendon lengthening surgery, were performed to achieve satisfactory clinical outcomes. All the patients resumed weight-bearing walking and daily exercises.

**Conclusion:**

Acute compartment syndrome and sequential complications could be managed using a number of medical procedures.

## Background

Acute compartment syndrome (CS) is a clinical complication that, although uncommon, is seen rather regularly in medical practice. Traumatic injuries such as fracture and crush-type injury are the main etiologies of CS, while other types of injury, such as ischemia-reperfusion injury after revascularization, posttraumatic arterial and venous thrombosis, tight splint, usages of tourniquet and shock trousers, snake bites and even drug injection, could also induce CS [1–4].

The foot and ankle are major weight-bearing structures in daily activities. Unfortunately, acute CS most often occurs in the anterior compartment of lower legs. For instance, the incidence rate of CS after tibia fracture could be as high as 30.4% [5, 6]. Lower leg CS induces ischemia and necrosis of involved leg muscles and nerves. Necrotic muscles may trigger systemic inflammatory responses, leading to organ failures and even death [4]. Furthermore, along with the imbalanced muscle strengths of extensors and flexors after surgical removal of the necrotic muscles, eventual fibrosis and retraction of the ischemic muscles result in foot and ankle deformities ranging from claw toes to multiplanar dislocations [7]. In addition, damaged and degenerated nerves of leg and foot largely impede patients’ mobility and wound healings [3]. For patients with recurrent ulcers and severe joint deformities they have to undergo amputation [1].

Due to severe edema and pain in lower extremity injuries, acute CS is difficult to diagnose which largely relies on the severity of the injury as well as experienced clinical examinations [8]. Therefore, current studies on CS mostly focus on early diagnosis and treatment. Early diagnosis is important to prevent catastrophic complications, including tissue necrosis, claw toe deformity, functional impairment, cavovarus deformities, neuromuscular injury, or joint contracture [9]. However, long-term wound management and sequential treatment of complications, particularly severe clubfoot deformity, are seldom reported [10].

In this study, we aimed to improve surgical management of acute CS. We reported the satisfactory recovery of 8 lower leg acute CS patients who were selected from 46 acute CS patients admitted at our hospital between 2008 and 2012 due to their severe muscle necrosis and clubfoot deformity. Our clinical findings showed that sequential treatment of wound management in the early stage and installation of Ilizarov external frame, as well as osteotomy fusion in the late stage of treatment, could effectively restore weight-bearing walking and daily exercises in CS patients.

## Methods

### Subjects

Between January 2008 and December 2012, 46 patients with lower leg CS and received fasciotomy were admitted to Burn Unit of Beijing Jishuitan Hospital for early wound treatment. All patients provided written consent and the study was approved by Ethics Committee of Jishuitan Hospital. Among 46 patients, 45 patients previously received fasciotomy to reduce tension at other hospitals, one patient had necrotic muscles all over his anterior and lateral compartment when admitted. Among the 46 patients, 38 patients accepted wound debridement surgeries and then wore corrective braces at Beijing Jishuitan Hospital to prevent foot drop in the following week, including 9 patients who received post-surgery joint capsule relaxation and tendon replacement.

Eight patients suffered complications such as secondary horseshoe ankle, metatarsophalangeal joint dislocation, foot varus and claw toes, which were too severe to be corrected simply via soft tissue treatment. These eight patients with severe CS complications were the subjects of this study. Their age range was 20–60 years (median age 37), including 7 males and 1 female. They were followed up for 3–5 years.

#### Protocols of CS treatment

### Systemic therapy

Antibiotics were administrated according to drug sensitivity test results to inhibit infection, and drugs were used to maintain normal function of the liver and the kidney. Anti-inflammatory drugs were used to inhibit systemic inflammatory reactions to prevent organ damages. Blood, plasma or albumin transfusion were applied if necessary.

### Wound management

Once the patients’ general conditions were stable, debridement was carried out as soon as possible to remove all the infected and necrotic muscles. This procedure was performed repeatedly if necessary. After thorough removal of necrotic tissues, wounds were closed via suturing, or in some cases via skin grafting.

### Prevention of foot drop and neuropathic ulcers

Within one week after the first wound debridement procedure, all eight patients wore foot drop correction braces (Beijing Yian Cubic Medical Tech.) until the application of Ilizarov external frame for clubfoot correction. When their ankles could reach the neutral position, the Ilizarov external frames were removed. The patients then wore the foot drop corrective braces while sleeping at night for further 3 months. When the patients practiced walking, their affected feet were further protected by orthopedic insoles to prevent neuropathic ulcers.

### Correction of clubfoot deformity by Ilizarov frame

Correction of foot deformity was carried out by traumatic orthopedic surgeons three months after the wound was stable (skin integrity, no ulcers or recurrence of infection). Under spinal or epidural anesthesia, the patient was positioned supine. Then, an assembled and sterilized Ilizarov external frame (non-limiting adaptive multidimensional arthrodesis orthotics, Beijing Yian Cubic Medical Tech.) was applied on the patient’s affected leg. Two Ilizarov rings were placed in proximal and distal ends of the tibia, respectively. Each ring was fixed by two Kirschner needles with a diameter of 2 mm. These two needles pinned through the tibia and formed an angle of about 50 degrees. Furthermore, they were fastened to a tension of 1000 N and secured on each ring by nuts. Additionally, two Ilizarov rings were connected with four adjustable equally-spaced threaded rods. Similarly, around the patient’s affected foot, an oval Ilizarov ring was fixed to the calcaneus via two Kirschner needles of the same size. The needles were secured to the rear half of the ring with a tension of 800 N. Another 2 mm Kirschner needle was attached to the front part of the oval ring with a tension of 800 N, penetrating all five metatarsal bones on their distal ends. Then, the Ilizarov ring in the distal end of the leg was connected to the ring surrounding the affected feet with adjustable thread rods.

On the third day after surgery, patients were instructed to take care of the implanted needles and practice interphalangeal joint dorsiflexion. By moving the nuts along the rods gradually towards the ends, the ring around the feet could pivot on the ankle and assist progressive ankle dorsiflexion.

### Other treatments

For ankle arthrodesis, 5 mm of cartilages and subchondral bones were excised between the distal end of the tibia and the trochlea of astragalus. Then, autogenous cancellous bones were implanted between two truncated bone ends, which were further fortified with an external fixator to press the bone ends together.

Claw toe deformity was corrected by proximal interphalangeal (PIP) joint resection arthroplasty, while claw toe deformity was corrected by flexor hallucis longus tendon lengthening surgery and metatarsophalangeal joint arthroplasty. Neuropathic ulcers were treated with wound dressings and with local flap grafting if necessary.

## Results

### Retrospective analysis of medical records of eight patients

Before admission to our hospital, five of eight patients were injured in car accidents, two by crush injuries, and one by sharp blades. Two patients had injuries in the right lower limbs and 6 patients had injuries in left lower limbs. Seven patients had bone fractures. All eight patients had damaged nerves, and peroneal nerve was badly severed in one patient. Five patients had impaired superficial and deep peroneal nerves due to secondary CS on their front and outer legs; two patients suffered superficial peroneal, deep and posterior tibial nerve injuries because of the involvement of multiple compartments, and one of them had sciatic nerve injury; four patients who had impaired blood vessels at the time of injury had vascular reconstruction surgeries at local hospitals; six patients had necrotic muscles of just the anterior and lateral compartments, while two other patients had necrosis in all lower leg muscles except for the gastrocnemius.

At local hospitals, six patients received fasciotomy to reduce tension within 12–24 h after injury, including one patent with additional fasciotomy due to insufficient tension reduction. In another patient, fasciotomy was carried out 2 days after injury. However, one patient did not receive fasciotomy immediately after injury and his anterior and lateral calf muscles were necrotic when he was admitted to a local hospital 6 months later.

### Wound management and complication treatment

Immediately after admission to our hospital, all patients had wound debridement 2–4 times to remove necrotic tissues completely. Wounds were sutured together for three patients, while skin grafting was utilized together with interrupted suturing on one side of the wound in the other five patients (Table 1).

**Table 1:** Clinical characteristics of leg injuries and early treatments

Patient	Age/gender	Cause of injury /Affected leg	Time span from injury to fasciotomy (h)	Fracture and treatment	Vascular injury and treatment	Damaged nerves	Necrotic compartments and treatment
1	36/M	Crush injury R	72	Femoral shaft/open reduction and internal fixation	Femoral artery (reconstruction)	Sciatic nerve, superficial and deep peroneal nerve and tibial nerve	A, L, PD 4 times of wound debridement, skin grafting and suturing
2	20/M	Crush injury L	13	Femoral condyle and capitulum fibulae/plaster slab		Peroneal nerve	A, PD, twice of wound debridement,skin grafting and suturing
3	25/M	Car accident L	18	Femoral shaft and Femoral condyle/open reduction and internal fixation	Popliteal artery (reconstruction)	Tibia nerve, superficial and deep peroneal nerve	A, L, PD, twice of wound debridement and suturing
4	49/M	Car accident L	24	Tibial plateau/ External fixator		Superficial and deep peroneal nerve	A, L, 4 times of wound debridement and suturing
5	60/F	Car accident L	14	Proximal end of the tibia and fibula/ External fixator		Superficial and deep peroneal nerve	A, L, 3 times of wound debridement and suturing
6	39/M	Car accident L	22	Femoral shaft/open reduction and internal fixation		Superficial and deep peroneal nerve	A, L, 4 times of wound debridement and suturing
7	29/M	Car accident L	16	Proximal end of the tibia and fibula /External fixator	Popliteal artery (reconstruction)	Peroneal nerve, Tibial nerve	A, L, 4 times of wound debridement and suturing
8	41/M	Sharp instrument injury R	N/A		Popliteal artery (reconstruction)	Tibial nerve, Superficial and deep peroneal nerve	A, L, twice of wound debridement and suturing

*M* Male, *F* Female, *R* Right, *L* Left, *H* Hour, *A* Anterior compartment, *L* Lateral compartment, *PD* Posterior compartment

All patients had various complications after discharge from the hospital. In two patients, neuropathic ulcers on the fifth metatarsal were improved following changing wound dressings. The clubfoot was rectified to the neutral position in Ilizarov external frames for all eight patients. When wearing Ilizarov external frames, four patients experienced claw toes, which were corrected by tendon lengthening and arthroplasty. Among all eight patients, six patients gradually resumed normal weight-bearing walking. The other two patients had severe clubfoot and received osteotomy and ankle fusion for permanent correction, and resumed walking.

### Representative case report

**Fig. 1:**
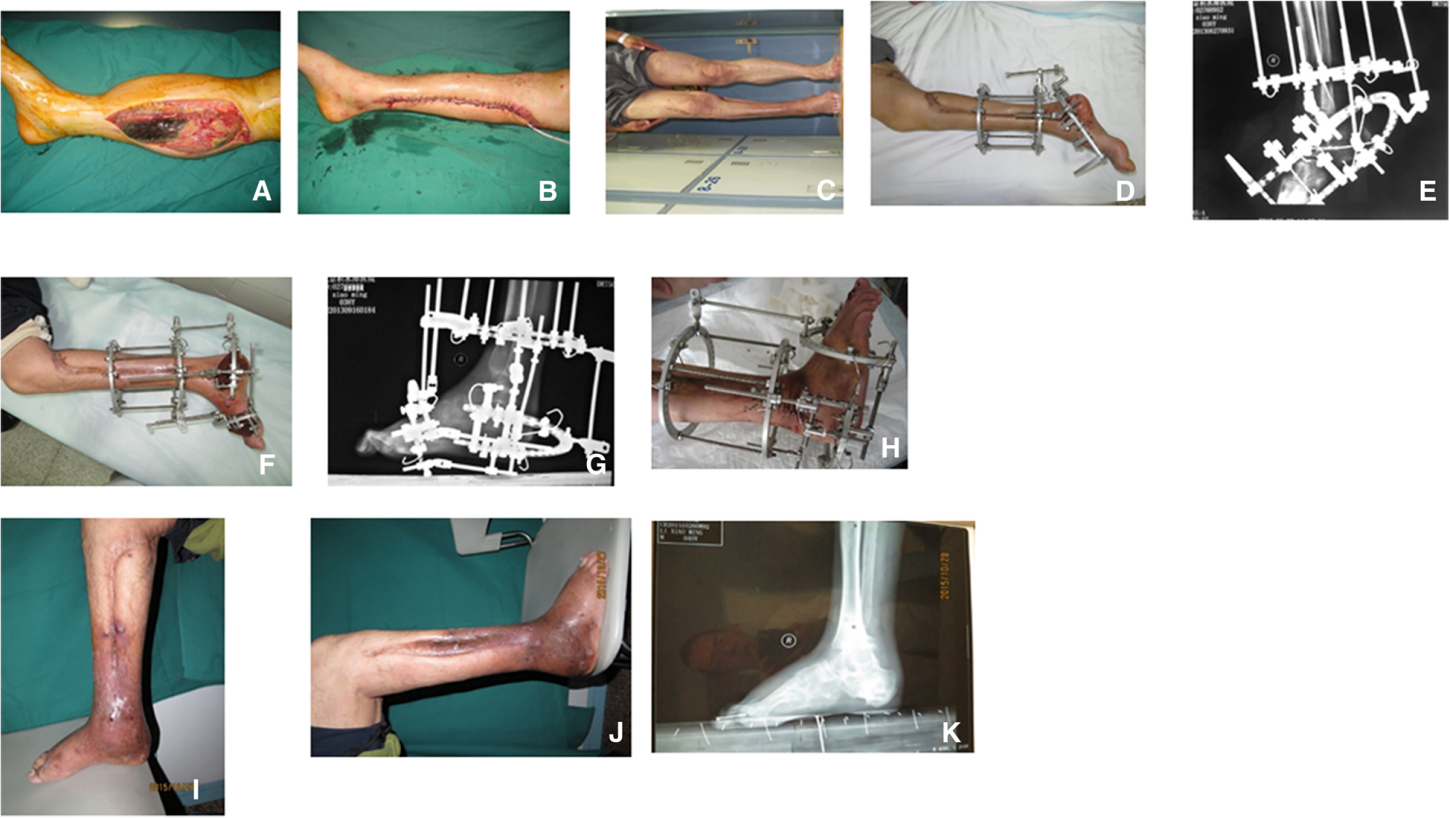
Representative images of one patient. **a**. The wound appearance after compartment fasciotomy of right lower limb. **b**. The wound was closed through skin suturation of right lower limb. **c**. The equinovarus deformity of right lower limb. **d**. The medial appearance after the application of Ilizarov fixator. **e**. X-ray image after the application of Ilizarov fixator. **f**. The ankle joint achieved 0。Dorsiflexion through the application of Ilizarov fixator. **g**. X-ray showed that ankle joint reached 0。Dorsiflexion. **h**. Image after the arthrodesis was performed. **i**. The equinovarus deformity was corrected completely (medial appearance). **j**. The equinovarus deformity was corrected completely (lateral appearance). **k**. X-ray showed that the equinovarus deformity was corrected completely

A 36-year-old male patient with lower limb CS was transferred to our burn unit on the third day post-trauma. Three days ago, crush injury occurred in his right thigh resulting in femoral shaft fractures, femoral vessels rupture and right sciatic nerve injury. The fracture was fixed with internal plates, and the ruptured femoral artery and vein was revascularized through vascular anastomosis 9 h after injury in his first visited hospital. Next day, he was transferred to another local hospital for fasciotomy to relieve edema of his right leg. He was then transferred to our hospital because of the complication on the third day after fasciotomy. The admission examination showed that his right leg swelled significantly compared to the left leg; the inner and outer sides of his leg each had an incision about 30 cm long to reduce tension; leg muscles bulged out and turned dark gray with no response to stimuli (Fig. 1a).

After admission, the patient underwent anti-infection and organ protection treatment. When he was stable, wound debridement was performed 4 times to clear all necrotic tissues with the exception of inner and outer sides of his surviving gastrocnemius heads. Wounds in the inner side of his leg were sutured, while the wound in the outer side was closed with a piece of skin graft (Fig. 1b). This patient used crutches to practice weight-bearing walking with the assistance of an orthopedic brace to prevent foot drop. One month later, neurological ulcers appeared on his fifth metatarsal base and were improved following changing wound dressings.

The patient was hospitalized again for Ilizarov external frame installation and clubfoot correction surgery 4 months after the wound was stable (Fig. 1c-e). The brace to prevent foot drop was then removed when the Ilizarov external frame was installed in order to stretch the muscles. However, this procedure induced claw toes on No.1–5 toes. Three months later, his ankle went back to neutral position. The patient was hospitalized again for Ilizarov frame adjustment, ankle fusion, lengthening flexor hallucis longus tendon of his first toe, metatarsophalangeal joint arthroplasty, and PIP joint resection of his No.2–5 toes (Fig. 1f-h). Six months later, Ilizarov frame was removed, and the patient resumed normal weight-bearing walking with the assistance of an anti-ulcer insole (Fig. 1i-k).

## Discussion

Acute CS of the lower leg is not widely reported, but its potential complications can develop after fractures, crush injury, or traumatic injury. Long-term devastating complications are known to seriously impede the mobility of the patients and the quality of life. CS related disabilities and even death were reported if the diagnoses and treatments were seriously delayed [11, 12].

All eight patients in this study suffered severe CS due to different traumatic injuries. They all had bone fractures and nerve injuries of various extents, damaged vascular and necrotic muscles, as well as severe clubfoot on their affected legs. Even after fasciotomy and other surgical treatment at local hospitals, they still experienced severe tissue dysfunctions and foot deformities, which required further wound treatment and multiple surgeries to achieve anatomical and functional recovery. In this study, we combined a series of treatments that covered every course of CS development and achieved satisfactory clinical outcomes in all patients.

The increase of compartment pressure induces CS, eventually leading to ischemic necrosis of muscles and nerves [13, 14]. Skeletal muscle is the dominant calf tissue and is most vulnerable to ischemia [4]. Labber et al. reported that 3, 4 and 5 h of ischemia led to necrosis in 2, 30 and 90% of leg muscles, respectively [15]. Conventional treatment after the detection of CS is immediate fasciotomy to reduce tension.

For CS patients with high-impact injuries caused by earthquakes, Huang et al. reported that more wound infections and amputations were associated with fasciotomy [16]. It was believed that as high as 15–25% of systemic complications were related to fasciotomy for CS patients with vascular damages on the lower extremity [17]. However, Faber et al. compared and analyzed the complications and efficacy of 612 cases of fasciotomy within (the early group) and after (the late group) 8 h following vascular restorations. The results indicated that the risks of amputations and infections in the early group were effectively lowered and the hospitalization duration was shortened in the early group compared to the late group [13]. Therefore, fasciotomy carried out long after the onset of CS may initiate undesired complications, which emphasizes the importance of early diagnosis and fasciotomy of CS.

In this study, six patients had fasciotomy 12–24 h after their affected legs got swollen (early group), while one patient had fasciotomy 2 days after the detection of CS and another patient was not diagnosed as CS at local hospital and received no fasciotomy (late group). Before fasciotomy, six patients in early group had necrotic anterior and outer lateral leg muscles, while two other patients in late group had necrotic muscles all over their affected legs except for gastrocnemius. Our clinical findings suggest that muscle necrosis could have been effectively prevented by early diagnosis as well as a timely fasciotomy.

Systemic support is beneficial to mitigate inflammatory reactions resulting from ischemic reperfusion, prevent organ complications, and improve the tolerance to surgery [4]. Both necrotic muscle tissues and their catabolic products could activate endogenous coagulate system and release inflammatory mediators [4]. All patients in this study had wound debridement 2–4 times to remove necrotic tissues. Suturing or skin grafting was applied to close the wounds. We speculate that these procedures largely contribute to satisfactory clinical outcomes. No systemic inflammatory response, infection or secondary organ damage was found throughout the course of treatment.

For patients with mild clubfoot, orthopedic braces are effective. In other cases, patients receive corrective surgeries, such as peroneus longus, brevis or posterior tibial muscle tendon displacement. In addition, arthrolysis on the rear side of ankle, including incision of posterior capsule film and Achilles tendon lengthening, could ease the deformity. At our hospital, most of the patients with acute CS had worn orthopedic braces during hospitalization. After being discharged from the hospital, the patients continued wearing the braces for another 3–6 months, allowing them to gradually resume daily walking and exercises. For those who had drop foot, similar programs of soft tissue treatment were carried out within 6 months after the closure of their wounds, which enabled them to walk and exercise.

Notably, in this study patients with severe complications all experienced at least one of secondary horseshoe ankle, metatarsophalangeal joint dislocation, foot varus and claw toes, which were too severe to be corrected simply via soft tissue treatment. Osteotomy fusion could restore clubfoot almost completely with high success rate and low recurrence [18]. However, this procedure is invasive, involving tarsal bone amputation and downsizing affected feet [18]. Furthermore, CS patients usually suffer vascular injuries and impaired soft tissues. All these concerns need to be taken into consideration before planning osteotomy fusion [7]. On the contrary, Ilizarov external frame is relatively noninvasive and less affected by impaired soft tissues, and facilitates tissue regeneration. As a result, deformed foot and ankle could be rearranged to normal position to correct the clubfoot [5–7].

In this study we summarized our experiences of managing eight patients with serious complications of acute CS. The sample size is relatively small, which is a limitation of this study. Further studies that enroll more patients with serious complications of acute CS are necessary.

## Conclusions

Lower leg CS is a serious complication that causes muscle, vascular and nerve damages as well as severe foot and leg deformities. We should stay alert to the onset of acute CS to promote early detection and timely fasciotomy of CS. The correction of deformations is important in the late stage of CS treatment. Our experience demonstrates that serious complications of acute CS could be managed.

## Abbreviations

CS: Compartment syndrome
PIP: proximal interphalangeal
